# Cytosolic sulfotransferase 1A1 regulates HIV-1 minus-strand DNA elongation in primary human monocyte-derived macrophages

**DOI:** 10.1186/s12985-016-0491-9

**Published:** 2016-02-24

**Authors:** Justine Swann, Jeff Murry, John A. T. Young

**Affiliations:** The Salk Institute for Biological Studies, 10010 North Torrey Pines Road, La Jolla, CA 92037 USA; University of California San Diego, 9500 Gilman Drive, La Jolla, CA 92093 USA; Gilead Sciences, 333 Lakeside Drive, Foster City, CA 94401 USA; Roche Innovation Center Basel, F.Hoffmann-La Roche Ltd, Grenzacherstrasse 124, 4070 Basel, Switzerland

**Keywords:** HIV-1, Monocyte-derived macrophages, Reverse transcription, Host factor, Sulfonation, Sulfotranserase

## Abstract

**Background:**

The cellular sulfonation pathway modulates key steps of virus replication. This pathway comprises two main families of sulfonate-conjugating enzymes: Golgi sulfotransferases, which sulfonate proteins, glycoproteins, glycolipids and proteoglycans; and cytosolic sulfotransferases (SULTs), which sulfonate various small molecules including hormones, neurotransmitters, and xenobiotics. Sulfonation controls the functions of numerous cellular factors such as those involved in cell-cell interactions, cell signaling, and small molecule detoxification. We previously showed that the cellular sulfonation pathway regulates HIV-1 gene expression and reactivation from latency. Here we show that a specific cellular sulfotransferase can regulate HIV-1 replication in primary human monocyte-derived macrophages (MDMs) by yet another mechanism, namely reverse transcription.

**Methods:**

MDMs were derived from monocytes isolated from donor peripheral blood mononuclear cells (PBMCs) obtained from the San Diego Blood Bank. After one week in vitro cell culture under macrophage-polarizing conditions, MDMs were transfected with sulfotranserase-specific or control siRNAs and infected with HIV-1 or SIV constructs expressing a luciferase reporter. Infection levels were subsequently monitored by luminescence. Western blotting was used to assay siRNA knockdown and viral protein levels, and qPCR was used to measure viral RNA and DNA products.

**Results:**

We demonstrate that the cytosolic sulfotransferase SULT1A1 is highly expressed in primary human MDMs, and through siRNA knockdown experiments, we show that this enzyme promotes infection of MDMs by single cycle VSV-G pseudotyped human HIV-1 and simian immunodeficiency virus vectors and by replication-competent HIV-1. Quantitative PCR analysis revealed that SULT1A1 affects HIV-1 replication in MDMs by modulating the kinetics of minus-strand DNA elongation during reverse transcription.

**Conclusions:**

These studies have identified SULT1A1 as a cellular regulator of HIV-1 reverse transcription in primary human MDMs. The normal substrates of this enzyme are small phenolic-like molecules, raising the possibility that one or more of these substrates may be involved. Targeting SULT1A1 and/or its substrate(s) may offer a novel host-directed strategy to improve HIV-1 therapeutics.

**Electronic supplementary material:**

The online version of this article (doi:10.1186/s12985-016-0491-9) contains supplementary material, which is available to authorized users.

## Background

Host cellular machinery is exploited to facilitate all steps of HIV-1 replication. A new paradigm in the treatment of HIV-1 infection is to target these so-called host-derived dependency factors, as exemplified by CCR5 coreceptor antagonists [1]. By contrast to the existing classes of direct-acting antivirals (DAAs) that target viral proteins, host-directed therapies may be less prone to the development of viral drug resistance and offer the potential for broader spectrum therapeutics [2, 3]. Therefore, there is currently a great deal of interest in better understanding the roles played by cellular factors and pathways during HIV-1 replication [4–8].

The cellular sulfonation pathway was first shown to play a key role in regulating HIV-1 infection at the level of cellular entry [9–13]. Previously, we uncovered another role for this pathway, demonstrating that it regulates retroviral transcription [14]. In that study, a forward genetic screen implicated two specific bi-functional 3′-phosphoadenosine-5′phosphosulfate (PAPS) synthetase enzymes, PAPSS1 and PAPSS2, as being important for retroviral replication [14]. These proteins catalyze two enzymatic steps to generate PAPS, the high-energy sulfonate-donor used in all cellular sulfonation reactions [15]. This specific effect was demonstrated using two different inhibitors of the sulfonation pathway: chlorate, a substrate analog of sulfate that blocks PAPS formation; and guaiacol, a sulfotransferase substrate mimic [14, 16]. In addition, we have recently shown that treatment with these chemical inhibitors significantly blocks HIV-1 reactivation from latency in a primary human CD4+ T cell model and in established cell lines where latency is maintained by diverse regulatory mechanisms [17].

**Fig. 1:**
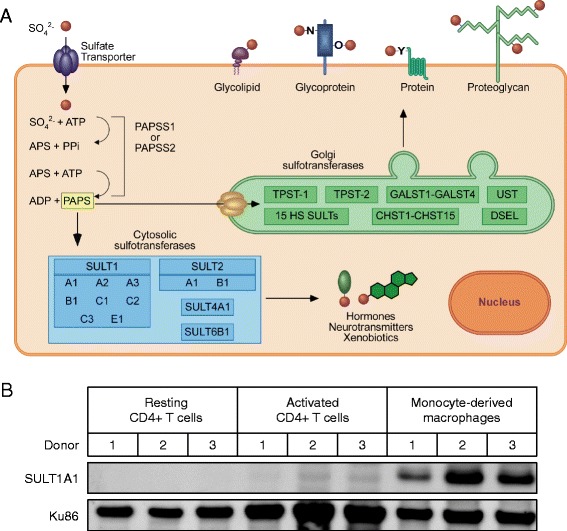
SULT1A1 is highly expressed in primary human monocyte-derived macrophages (MDMs). **a** The cellular sulfonation pathway. The first step of the cellular sulfonation pathway involves import through a sulfate transporter of a sulfate ion that is then used as a substrate by either 3′-phosphoadenosine-5′phosphosulfate (PAPS) synthetase enzymes PAPSS1 or PAPSS2. These proteins catalyze two enzymatic steps to generate PAPS, the high-energy universal sulfonate-donor from sulfate and two molcules of ATP. PAPS can be transported across the Golgi membrane and used by the Golgi sulfotransferases to generate sulfonated proteins, glycoproteins, glycoproteins, glycolipids, and proteoglycans. Alternatively, PAPS can be used by cytosolic sulfotransferases (SULTS) to sulfonate small molecules such as hormones, neurotransmitters, and xenobiotics. **b** Human CD4+ T cells and CD14+ monocytes were isolated from donor PBMCs by magnetic bead isolation. Resting CD4+ T cells were lysed directly after separation, and the remaining CD4+ T cells were activated using CD3/CD28 beads for three days. Monocytes were cultured for 7 days in the presence of 20 ng/ml M-CSF, were lysed, subjected to gel electrophoresis, and immunoblotting was performed to detect SULT1A1 or the loading control Ku86 protein

The cellular sulfonation system consists of a family of sulfotransferase enzymes that are responsible for sulfonate transfer within the cell [18]. These proteins catalyze the transfer of a sulfuryl group (SO_3_^−^) from PAPS to a hydroxyl or amino-group on an acceptor molecule. Sulfotransferases are organized into two main sub-families: the Golgi and cytosolic sulfotransferases. The Golgi sulfotransferases are membrane bound enzymes that sulfonate cell surface proteins, carbohydrates, proteoglycans, and glycoproteins, while the cytosolic sulfotransferases are cytoplasmic enzymes that sulfonate endogenous hormones, neurotransmitters, and small metabolites as well as exogenous xenobiotics (Fig. 1a). Cell surface sulfonation is necessary during normal homeostatic processes such as lymphocyte homing and cellular signaling [19–22]. Cytoplasmic sulfonation of metabolites by the cytosolic sulfotransferases (SULTs) generally leads to their inactivation, detoxification, and/or bioactivation [23–27]. Here we show that one of these cytosolic sulfotransferases, SULT1A1, regulates HIV-1 reverse transcription in MDMs, increasing our knowledge of the roles played by cellular factors in regulating HIV-1 reverse transcription.

## Results

### Human cytosolic sulfotransferase 1A1 is highly expressed in primary human monocyte-derived macrophages

To determine if any of the 12 known cytosolic SULTs might play a role in regulating HIV-1 infection, we first compared their relative expression levels in the two physiologically relevant cell types, primary human CD4+ T cells and primary monocytic cells. We employed the publically available mRNA expression database Bio-GPS [28]. This analysis revealed a strikingly high level of SULT1A1 mRNA in CD14+ monocytes as compared to other other cell types including CD4+ T cells (Additional file 1: Figure S1). Although SULT1A1 was previously found to be important in tissues such as the liver, brain, kidney, and gastrointestinal tract, little or nothing is known about its role in immune cells [29–31]. MDM-selective expression was confirmed by immunoblot analysis of protein lysates obtained from primary human CD4+ T cells and MDMs. In agreement with the mRNA expression data, SULT1A1 was highly expressed in MDMs. However, SULT1A1 protein expression was undetectable in resting or activated CD4+ T cells (Fig. 1b).

### SULT1A1 knockdown decreases retroviral infection in MDMs

**Fig. 2:**
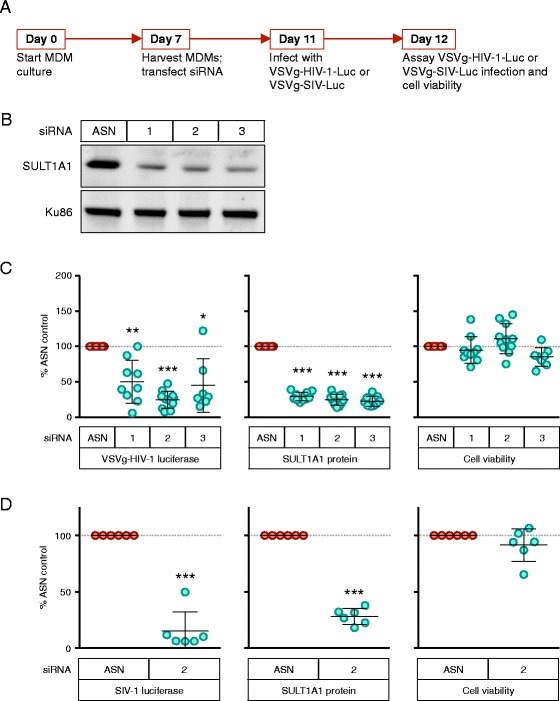
SULT1A1 knockdown is associated with decreased viral gene expression following infection of MDMs with VSV-G pseudotyped HIV-1 and SIV vectors. **a** Schematic showing experimental timeline. Briefly, CD14+ monocytes were isolated from human donor PBMCs using positive selection with magnetic beads. Monocytes were differentiated into MDMs and on day 7 were electroporated with siRNA and plated at 1.5 × 10^4^ MDMs per well in a 48 well plate. After 96 h, protein knockdown was confirmed by immunoblot and cells were infected with 100 μl (corresponding to 164 ng p24 HIV-1 and 234 ng p27 SIV viral vectors) of the indicated virus. Luciferase and cell viability measurements were determined at 24 h post-infection. **b** Representative immunoblot showing SULT1A1 knockdown 96 h post transfection with SULT1A1 siRNAs (1–3) and AllStars Negative siRNA Control (ASN), Qiagen. SULT1A1 expression is compared to endogenous Ku86 used as a loading control. Results from one representative donor are shown. SULT1A1 expression was generally decreased by 70–80 % with siRNA treatment compared to the control ASN siRNA (as shown in Fig. 2c, middle panel). **c** HIV-1 luciferase reporter expression (*left panel*), SULT1A1 protein expression (*middle panel*), and cell viability of MDMs (*right panel*) were measured 24 h after infection with the VSV-G pseudotyped NL43-Luc HIV-1 vector. Results shown are from 6 donors assayed twice. All values were compared to ASN control. **d** SIV-1 luciferase reporter expression (left panel), SULT1A1 protein expression (*middle panel*), and cell viability (*right panel*) for MDMs 24 h post infection with VSV-G-pseudotyped SIVagm-Luc. Mean and SD shown, *** *p* < 0.0005 ** *p* < 0.005 * *p* < 0.05 one sample t test. Results shown are from 6 donors assayed twice. Samples with <60 % SULT1A1 knockdown and/or <65 % cell viability were not used in the analysis. All values were compared to ASN control

To test if SULT1A1 plays a role during retroviral infection in MDMs, siRNA knockdown was used to reduce the levels of this protein (Fig. 2a). Three distinct SULT1A1-targeting siRNAs (#1–3) each reduced SULT1A1 protein levels by 70–80 % at 96 h post-transfection as compared to a control (scrambled sequence) siRNA (Fig. 2b, c middle panel). Each of the 3 SULT1A1 siRNAs also reduced the levels of infection seen with a VSV-G-pseudotyped HIV-1 NL43 virus vector as judged by monitoring expression of the firefly luciferase reporter enzyme from the viral genome [32]. Knockdown of SULT1A1 expression with SULT1A1 siRNAs 1, 2, or 3 resulted in a decrease in HIV-1 reporter gene expression by 50 % (*p* < 0.005), 76 % (*p* < 0.0005), and 57 % (*p* < 0.005), respectively (Fig. 2c, left panel). None of the siRNAs used significantly impacted cell viability (Fig. 2c, right panel). The fact that all three SULT1A1-directed siRNAs reduced viral gene expression following infection strongly argues against an indirect off-target effect being responsible for this effect.

To test if SULT1A1 could also influence infection by the related simian immunodeficiency virus, SULT1A1 siRNA-treated and control siRNA-treated MDMs were infected with a VSV-G-pseudotyped SIVagm virus vector encoding firefly luciferase (Fig. 2d) [33]. As for HIV-1, RNAi-mediated knockdown of SULT1A1 directly correlated with a significant reduction in expression of the virus-encoded reporter enzyme, with an average of 85 % inhibition (*p* < 0.0005) (Fig. 2d, left panel).

**Fig. 3:**
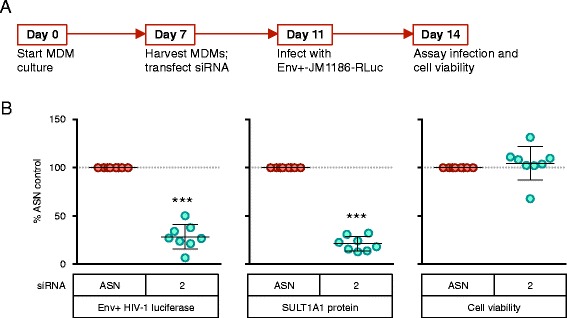
SULT1A1 knockdown is associated with decreased viral gene expression following infection of MDMs with a replication-competent HIV-1 vector. **a** The same method was used as in Fig. 2a, however 100 μl (corresponding to 66.3 ng p24) HIV-1 Env + JM1186-Rluc virus was added to the cells at day 11 and luciferase and cell viability was assayed at 72 h post-infection. **b** HIV-1 luciferase reporter expression, SULT1A1 protein expression, and cell viability of MDMs 24 h post infection with HIV-1 Env + JM1186-RLuc.. Mean and SD shown, *** *p* < 0.0005 ** *p* < 0.005 * *p* < 0.05 one sample t test. Results shown are from 6 donors assayed twice. Samples with <60 % SULT1A1 knockdown and/or <65 % cell viability were not used in the analysis. All values were compared to ASN control

To further validate the role of SULT1A1 during HIV-1 infection, we next investigated the impact of knocking down expression of this protein on infection of MDMs by replication-competent HIV-1. MDMs that were transfected with control siRNA or with SULT1A1-siRNA#2 were challenged with the replication-competent CCR5-tropic HIV-1 Env + JM1186 virus encoding Renilla luciferase [34, 35] and luciferase expression was assayed three days post infection (Fig. 3a). SULT1A1 knockdown was associated with a significant inhibition of virus-encoded luciferase expression in MDMs derived from multiple donors without affecting cell viability (Fig. 3). Transfection of these cells with SULT1A1 siRNA #2 resulted in an average 72 % decrease in HIV-1 reporter expression compared to control (*p* < 0.0005) (Fig. 3, left panel). Taken together, these results demonstrate that SULT1A1 is important for efficient virus gene expression following infection of MDMs with single cycle HIV-1 and SIV vectors and with replication-competent HIV-1.

### SULT1A1 regulates HIV-1 reverse transcription

To determine the step in HIV-1 replication that is influenced by SULT1A1, we monitored the effect of SULT1A1 siRNA #2 on early steps of virus replication at 24 h post-infection. The VSV-G-pseudotyped HIV-1 vector was used for these studies to avoid multiple cycles of virus infection confounding the interpretation of the results. Quantitative PCR methods were used to monitor viral DNA and RNA products, focusing specifically upon spliced viral RNAs. These RNAs are produced *de novo* in infected cells and thus, are clearly distinguished from the large amounts of input unspliced viral genomic RNA that are present in these cells due to efficient virus uptake. Immunoblotting was used to monitor the expression levels of two independent viral proteins (Vpu and Vif), that are produced from spliced HIV-1 mRNA transcripts.

**Fig. 4:**
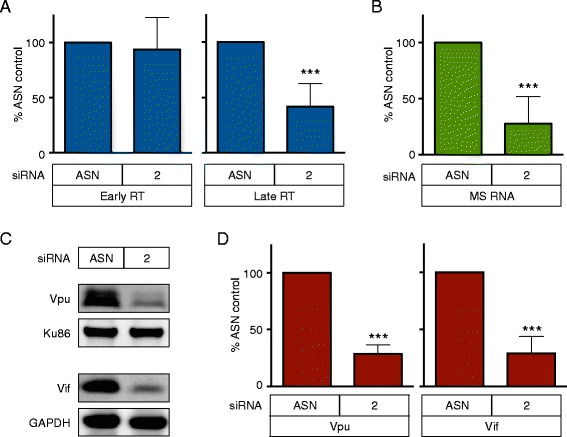
SULT1A1 regulates HIV-1 reverse transcription. **a** MDMs were treated with control siRNA or SULT1A1-specific siRNA and subesequently challenged with VSV-G pseudotyped NL43-Luc HIV-1 vector. DNA was isolated 24 h post-infection and qPCR was performed using primers that detect early RT DNA products or late RT DNA products compared to the cellular PBGD gene as an endogenous control. The levels of early and late RT products were normalized to the ASN siRNA control. Results shown are from MDMs derived from 6 donors tested twice. **b** MDMs were transfected with SULT1A1 siRNA 2 or ASN siRNA and total cellular RNA was isolated 24 h post infection with VSV-G pseudotyped NL43-Luc HIV-1 vector. qPCR using primers specific for HIV-1 multiply spliced mRNA (MS RNA, forward and reverse primers span the first and second exons of Tat/Rev, respectively) was performed, and relative MS RNA (normalized to GAPDH) was then normalized to ASN siRNA control. Results shown are from MDMs derived from 6 donors tested twice. **c** Representative immunoblot showing HIV-1 Vpu and Vif protein levels compared to endogenous Ku86 or GAPDH loading control, respectively, from protein lysate collected 48 h post infection with VSV-G pseudotyped NL43-Luc HIV-1 vector pre-treated with either ASN siRNA control or SULT1A1 siRNA 2. **d** Quantitative immunoblot analysis using Image Studio software of Vpu and Vif as shown in Fig. 4c. Mean and SD shown, *** *p* < 0.0005 ** *p* < 0.005 * *p* < 0.05 one sample t test. Results shown are from 6 donors assayed twice. Samples with <60 % SULT1A1 knockdown and/or <65 % cell viability were not used in the analysis. All values were compared to ASN control

These studies revealed that SULT1A1 had no effect upon the levels of early reverse transcription products (defined as those generated prior to minus-strand DNA transfer) (Fig. 4a, left panel). By contrast, knockdown of SULT1A1 was associated with a reduction (58 %, *p* < 0.0005) in the levels of late reverse transcription products (defined here as those generated subsequent to minus-strand DNA transfer) (Fig. 4a, right panel). Consistently, SULT1A1 knockdown in MDMs was associated with a 72 % reduction in levels of HIV-1 multiply spliced RNA (forward and reverse primers span the first and second exons of Tat/Rev, respectively (*p* < 0.0005) (Fig. 4b). Furthermore, SULT1A1 knockdown was also correlated with a 70 % reduction in the levels of both the HIV-1 Vif and Vpu proteins (*p* < 0.0005) (Fig. 4c-d). Taken together, these results indicate that the predominant effect of SULT1A1 on HIV-1 replication is at the level of viral DNA synthesis.

### SULT1A1 influences the kinetics of HIV-1 minus-strand DNA elongation

**Fig. 5:**
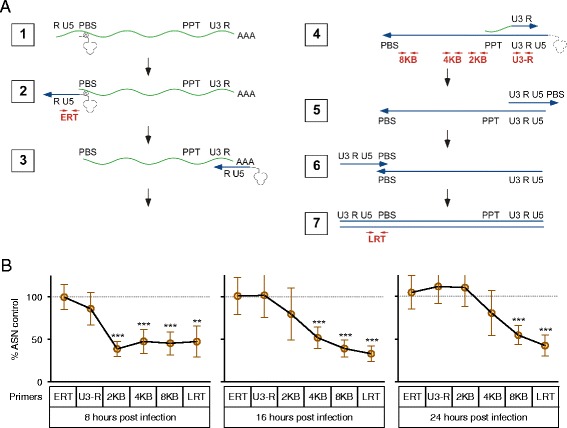
SULT1A1 influences the kinetics of minus-strand DNA elongation. **a** The step during HIV-1 reverse transcription that SULT1A1 regulates was investigated by quantitative real-time PCR analysis with the indicated primer sets. The stages of reverse transcription are shown: 1. Viral genomic RNA; 2. Minus-strand DNA initiation; 3. Minus-strand DNA transfer; 4. Minus-strand DNA elongation and plus-strand DNA initiation; 5 and 6. Plus-strand DNA transfer; 7. Plus-strand DNA elongation. **b** MDMs were treated with control or SULT1A1-specific siRNA and challenged with VSV-G pseudotyped NL43-Luc HIV-1 vector. Total cellular DNA was isolated at 8, 16, or 24 h post infection. Quantitative real-time PCR was performed to measure the relative abundance of DNA products corresponding to specific steps during the process of reverse transcription using primers indicated in Fig. 5a. Results shown are from MDMs derived from 6 donors assayed once. Mean and SD shown, *** *p* < 0.0005 ** *p* < 0.005, one sample t test

In order to determine the specific stage of HIV-1 reverse transcription that is influenced by SULT1A1, MDMs were transfected with SULT1A1-specific or control siRNA prior to infection with VSV-G pseudotyped NL43-Luc HIV-1 vector and DNA was isolated at different time points post-infection (ranging from 8–24 h). The relative abundance of different length reverse transcription products was then measured using a series of oligonucleotide primers that were used to amplify different regions of HIV-1 DNA (labeled in order of appearance as follows: ERT, U3-R, 2 KB, 4 KB, 8 KB, LRT (Fig. 5a).

Consistent with our previous result (Fig. 4), the abundance of early reverse transcription products (ERT and U3-R) was largely the same in both SULT1A1 siRNA transfected and control transfected cell populations (Fig. 5b all panels). By contrast, there was a marked reduction of DNA products longer than 2 kb in length at 8 h post-infection in cells deficient in SULT1A1 (Fig. 5b, left panel). The defect in 2 kb products was less pronounced at 16 h post-infection and was not evident at 24 h post-infection (Fig. 5b). Similarly the defect in the level of 4 kb products was less pronounced at 24 h as compared with 16 h post-infection. The defect seen in the levels of longer 8 kb and LRT products was not overcome in the 24-h time frame. Taken together, these results show that SULT1A1 knockdown is associated with a decreased progression of reverse transcription, a result that is consistent with this enzyme playing an important role during the kinetics of viral minus strand DNA elongation.

## Discussion

This study demonstrates that the cellular sulfotransferase SULT1A1 regulates HIV-1 reverse transcription in primary human monocyte-derived macrophages (MDMs). We showed that SULT1A1 is highly expressed in MDMs and that RNAi-mediated depletion of SULT1A1 in these cells results in a significant reduction in the kinetics of minus-strand DNA elongation during HIV-1 reverse transcription.

The synthesis of HIV-1 proviral DNA from the viral RNA genome is heavily regulated by cellular factors; however, only a small number of these factors have been well characterized to date [36–39]. These proteins include SAMHD1, which blocks reverse transcription in myeloid cells and resting CD4+ T cells by hydrolyzing dNTPs and/or degrading viral RNA [40–43], and APOBEC3G, which deaminates cytidine to uridine resulting in hypermutation during DNA synthesis [44, 45]. In addition, other factors have been shown to affect the kinetics of viral DNA synthesis [5]. Thus, the studies described in this report extend our understanding of the roles of cellular factors in regulating this key step of retroviral replication. This novel role for SULT1A1 in the regulation of retroviral reverse transcription also adds to our growing knowledge about the diverse roles that the sulfonation pathway plays in the retroviral life cycle. For example, tyrosylprotein sulfotransferases 1 and 2 (TPST1 and TPST2) are Golgi sulfotransferases that sulfonate N-terminal tyrosine residues of CCR5 and enable efficient cellular entry of R5-tropic viruses [9, 10, 13]. It has also been demonstrated that HIV-1 binds to sulfonated proteoglycans on cell surfaces [11, 12]. In addition, we previously showed that PAPSS deficiency or treatment of cells with the sulfonation pathway inhibitors chlorate and guaiacol blocked HIV-1 *de novo* gene expression from the viral LTR promoter, and more recently we showed that these inhibitors also block HIV-1 reactivation from latency [14, 17]. Taken together, these observations demonstrate the importance of the sulfonation pathway at multiple steps of HIV-1 replication. It will be important for future studies to determine which sulfotranserase(s) regulate HIV-1 infection and reactivation from latency in CD4+ T cells, as SULT1A1 does not appear to be expressed at the protein level in these cells and control has been demonstrated at level of transcription, not reverse transcription, upon treatment with chlorate and guaiacol.

*SULT1A1* is highly polymorphic within the human population, with both genetic polymorphisms and copy number variation conferring different levels of enzymatic activity [46–48]. Moreover, *SULT1A1* variation has been linked to a number of diseases such as cancer [49–52], heart disease [53], and inflammatory bowel disease [54]. Consequently, we are now seeking to determine if there is a correlation between *SULT1A1* variability and HIV-1 susceptibility and/or AIDS disease progression.

Further investigation will be aimed at determining if SULT1A1 acts on HIV-1 through a substrate-dependent or -independent mechanism. It is possible that SULT1A1 may act independently of substrate by directly modifying viral proteins (such as reverse transcriptase). If the sulfonation of a specific SULT1A1 substrate is required, on the other hand, then identification of that substrate will be critical for understanding the underlying mechanism involved.

## Conclusions

In summary, we demonstrated that a human cytosolic sulfotransferase, SULT1A1, regulates HIV-1 reverse transcription in human monocyte-derived macrophages (MDMs). We showed that SULT1A1 is highly expressed in primary human monocytes and MDMs. RNAi-knockdown of SULT1A1 in MDMs leads to a substantial decrease in infection by both pseudotyped and replication-competent HIV-1 vectors, as well as by a SIVagm vector. Quantitative PCR analysis revealed that this effect is associated with a defect in minus-strand DNA elongation during HIV-1 reverse transcription. These results support the idea that SULT1A1 is a novel HIV-1 host factor in MDMs, and suggest that targeting SULT1A1 or its substrate may lead to improved HIV-1 therapies.

## Methods

### Reagents

AllStars Negative control and SULT1A1 Flexitube siRNAs (sequences provided in Additional file 2: Table S1) were obtained from Qiagen, reconstituted at 20 μM in water, and stored at −20 °C until use. Cell viability was assayed using Cell Titer-Glo reagent and luciferase activity was measured using Bright-Glo reagent according to the manufacturer’s instructions (Promega).

### SULT mRNA expression analysis

The expression level for each cytosolic sulfotransferase in CD4+ T cells and CD14+ monocytes was derived from publically available expression data from BioGPS [28], and normalized to the median expression of that sulfotransferase in all tissues tested.

### Peripheral blood mononuclear cells

Human donor buffy coats and LRS-WBC (white blood cells isolated in the Leuko-Reduction system via Terumo BCT Trima Automated Collection System) samples were collected from anonymous healthy donors and obtained from the San Diego Blood Bank. Written informed consent for the use of buffy coats and LRS samples for research purposes was obtained from the donors by the San Diego Blood Bank. Samples are routinely tested for the presence of HIV-1 antibodies and all samples used in the study are confirmed HIV-1 negative. Peripheral blood mononuclear cells (PBMCs) were isolated from these samples by Ficoll density gradient centrifugation. Briefly, buffy coats and LRS samples were diluted in RPMI, layered over Ficoll (Ficoll-Paque Plus, GE Healthcare), and centrifuged at 1850 rpm for 25 min without brakes. Cells at the interface were removed and washed twice in RPMI. Platelets were then removed by a final spin at 800 rpm without brakes. The cells were counted and resuspended at a density of 25x10^6^ cells/ml in FBS supplemented with 10 % DMSO, transferred to −80 °C at least overnight, and then to liquid nitrogen for long-term storage.

### CD4+ T cells

PBMCs were thawed in RPMI, washed, and CD4+ T cells were isolated using CD4+ negative selection magnetic bead isolation (EasySep Human CD4+ T cell enrichment kit, StemCell Technologies). Resting CD4+ T cells were lysed directly after separation, and the remaining CD4+ T cells were activated using CD3/CD28 beads for three days (Dynabeads Human T-Activator CD3/CD28, Life Technologies). CD4+ T cells were resuspended at 2x10^6^ cells/100 μl 1× LDS lysis buffer (Invitrogen), diluted 1:3, and 10 μl lysate was loaded onto a gel 4–12 % Bis-Tris gel for immunoblot analysis.

### Monocyte-derived macrophages

PBMCs were thawed in RPMI, washed, and CD14+ monocytes were isolated using CD14 positive selection magnetic cell sorting according to the manufacturer’s instructions (Easy Sep Human CD14 Positive Selection kit, StemCell Technologies). CD14+ monocytes were plated at 5x10^6^ cells per 10 cm polypropylene petri dish (Fisher) in RPMI 1640 medium (Gibco-BRL) supplemented with 10 % Human AB Serum (Sigma), 50 U/ml penicillin, 50 μg/ml streptomycin, 10 mM HEPES, sodium pyruvate, and 20 ng/ml recombinant human M-CSF (Peprotech). Fresh media without cytokine was added after 3–4 days, and by day 7 the MDMs were fully differentiated.

### siRNA transfection

MDMs were washed with PBS, treated with Accutase (Stem Pro Accutase Cell Dissociation Reagent, Life Technologies) at 37 °C for 30 min, and dissociated by gentle pipetting. Cells were transfected with siRNA using the NEON electroporation system (Life Technologies) according to the manufacturer’s instructions. Briefly, 1.5×10^5^ MDMs were mixed with 3 μl 20 μM siRNA in a 10 μl final reaction volume, electroporated with 2 pulses of 1500 V for 20 ms, and then transferred to 500 μl pre-warmed RPMI 10 % human serum without antibiotics in a 48 well plate and allowed to recover for 4 days. At 4 days post transfection, samples were assayed for protein knockdown by immunoblot. Samples with <60 % SULT1A1 knockdown and/or <65 % cell viability were not used in the analysis.

### Viruses and infection

The HIV-1 NL43-Luc and SIVagm plasmids were obtained from Ned Landau [32, 33]. HIV-1 Env + JM1186-Rluc plasmid was made by Sumit Chanda’s lab by cloning the V3 loop of Gp120 from isolate 92TH014 [35] and Renilla luciferase reporter into pBR-NL43-IRES-eGFP [34]. VSV-G pseudotyped NL43-Luc HIV-1 and Env + JM1186-Rluc HIV-1 viruses were produced by transient transfection of 293 T cells by the Salk viral vector core, and VSV-G-pseudotyped SIVagm-Luc was produced in a similar method as previously described [55]. HIV-1 and SIVagm viruses were assayed for p24 or p27 content, respectively, by p24 or p27 antigen capture ELISA according to the manufacturer’s instructions (Zeptometrix).

For MDM infection experiments, media was removed 96 h post transfection with siRNA, replaced with 100 μl virus (corresponding to 164 ng p24 VSV-G pseudotyped NL43-Luc HIV-1, 66.3 ng p24 Env + −JM1186-Rluc HIV-1, 234 ng p27 VSV-G pseudotyped SIVagm-Luc) diluted in 400 μl fresh RPMI 10 % human serum, and cells were spinoculated at 1200 × g for 60 min. Viral reporter expression and cell viability was assayed 24 h post infection for VSV-G-pseudotyped viruses, and 3 days post infection for replication competent HIV-1.

### Immunoblot and protein quantitation

Cells were lysed in 1× LDS sample buffer (Invitrogen) and stored at −20 °C until use. Samples were thawed on ice, incubated at 95 °C for 5 min, and centrifuged for 3–5 min before loading. Precision Plus Protein Dual Color Standards (Bio-Rad) or Novex Sharp Pre-stained standards (Invitrogen) were used as protein standards, and 4–12 % Bis-Tris gels (Invitrogen) were used for gel electrophoresis. Protein was transferred onto PVDF membrane (Millipore Immobilon-FL) using BioRad wet transfer apparatus at 100 V for 1 h at 4 °C. Membranes were blocked with Casein blocking buffer (Bio-Rad) for at least 1 h at room temperature, then incubated with primary antibody diluted in 0.1 % Casein/0.2× PBS + 0.1 % Tween-20 overnight at 4 °C. Primary antibodies used in the study include mouse anti-human SULT1A1 mAb (clone 638708, R&D Systems) used at 1:2000 dilution, rabbit anti-HIV-1 Vif (clone A319, NIH AIDS Reagent program) used at 1:500, rabbit anti-HIV-1 Vpu (clone ab81532, Abcam) used at 1:1000 dilution, mouse anti-human Ku-86 mAb (clone B-1, Santa Cruz Biotechnology) used at 1:500 dilution, and anti-human mouse GAPDH (clone 6C5, Abcam) used at 1:5000. Membranes were washed three times with 1× PBS/ 0.1 % Tween-20, incubated with Alexa-Fluor 680-conjugated secondary antibody diluted in 0.1 % Casein 0.2× PBS 0.1 % Tween-20 and 0.01 % SDS at room temperature for at least 1 h. Secondary antibodies used in the study include goat anti-mouse Alexa-Fluor 680 IgG (H + L) (clone A21057, Invitrogen), and donkey anti-rabbit Alexa-Fluor 680 IgG (H + L) (clone A10043, Invitrogen). Membranes were washed three times with 1× PBS/ 0.1 % Tween 20, and imaged using the Licor Odyssey system. Protein expression was quantified using Image Studio software (Licor).

### Detection of nucleic acids by real-time quantitative PCR

Total RNA was prepared using Qiazol lysis reagent (Qiagen) and miRNeasy RNA isolation kit (Qiagen) according to the manufacturer’s instructions. cDNA was synthesized using Quantitect Reverse Transcription kit (Qiagen) according to the manufacturer’s instructions. DNA was isolated from cells using a DNeasy Blood and Tissue Kit (Qiagen) according to the manufacturer’s instructions. Quantitation of ERT, LRT, and PBGD was performed using primers and probes diluted in TaqMan universal PCR master mix (Invitrogen). Amplification of other DNA products was monitored using SYBR green fluorescence (Invitrogen). Real-time PCR was performed using the ViiA 7 Real-time PCR system (Life Technologies). Standard curves were generated for all primers and efficiencies were found to be equivalent. Relative expression was calculated using the delta CT method as previously described [56]. All primer and probe sequences used in the study are included in Additional file 2: Table S1.

## Additional files

Additional file 1: Figure S1. SULT1A1 is highly expressed in monocytes. The expression level for each cytosolic sulfotransferase in CD4+ T cells and CD14+ monocytes was derived from publically available expression data from BioGPS and normalized to the median expression of that sulfotransferase in all tissues tested. (PDF 26 kb) Additional file 2: Table S1. Primer and siRNA sequences. DNA and RNA primer sequences used in the study are listed from 5′ to 3′. siRNAs are listed with the siRNA name, Qiagen ID, and sequences from 5′ to 3′. (XLSX 43 kb)

## Abbreviations

MDM: monocyte-derived macrophage
PBMC: peripheral blood mononuclear cell
