# Supplementary figures and images for: Cytosolic sulfotransferase 1A1 regulates HIV-1 minus-strand DNA elongation in primary human monocyte-derived macrophages
Source: Virol J. 2016 Feb 24;13:30. doi: 10.1186/s12985-016-0491-9 (PMC4765207; doi:10.1186/s12985-016-0491-9)

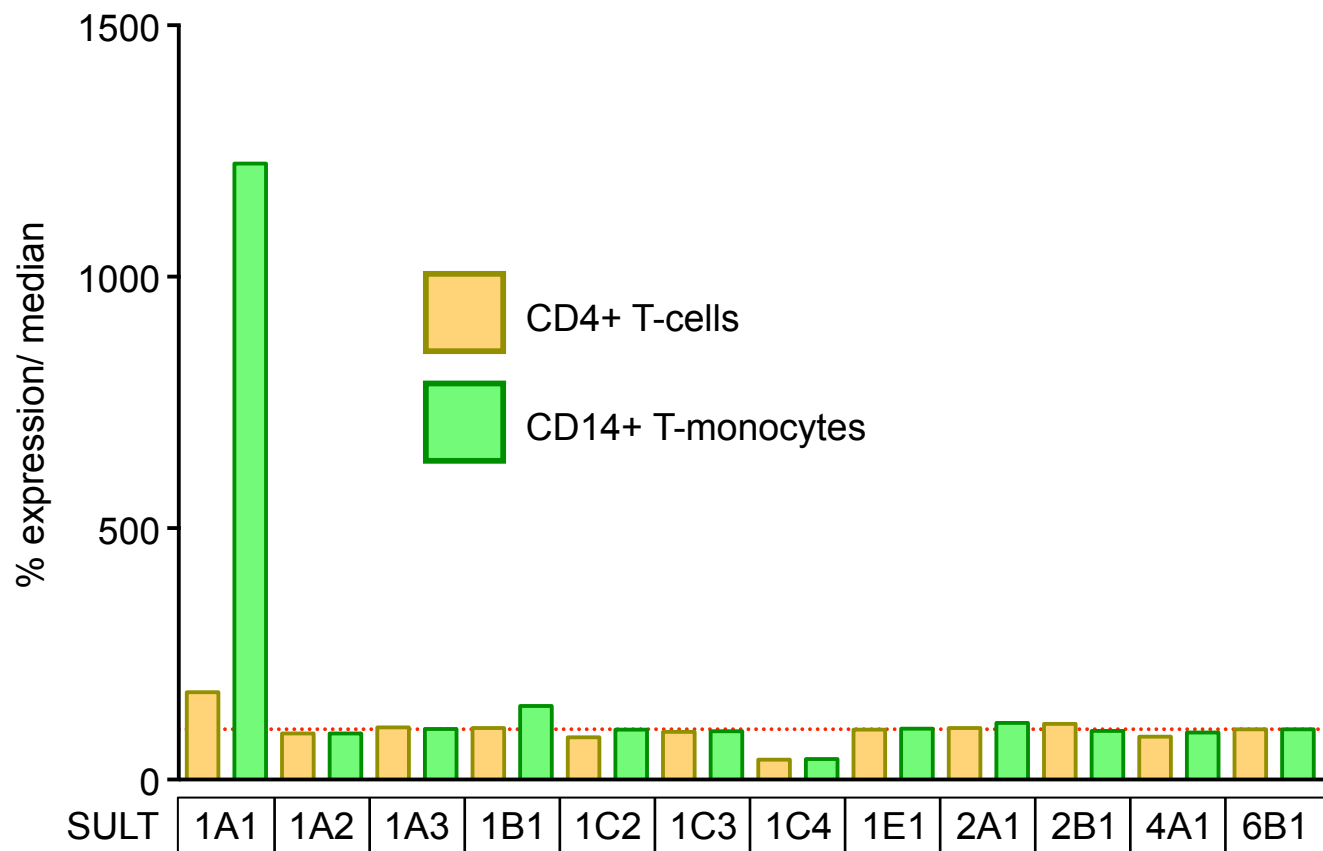

Supplement: Additional file 1: Figure S1. — SULT1A1 is highly expressed in monocytes. The expression level for each cytosolic sulfotransferase in CD4+ T cells and CD14+ monocytes was derived from publically available expression data from BioGPS and normalized to the median expression of that sulfotransferase in all tissues tested. (PDF 26 kb) [file 12985_2016_491_MOESM1_ESM.pdf]
